# Association between dipsacus saponin VI level and diversity of endophytic fungi in roots of *Dipsacus asperoides*

**DOI:** 10.1007/s11274-019-2616-y

**Published:** 2019-02-18

**Authors:** Anhui Gong, Tao Zhou, Chenghong Xiao, Weike Jiang, Yongqiang Zhou, Jinqiang Zhang, Qing Liang, Changgui Yang, Wei Zheng, Chenggang Zhang

**Affiliations:** 1 Experimental Center, Guiyang University of Chinese Medicine, Guiyang, 550025 China; 2 Department of Molecular Biology Laboratory, Guiyang University of Chinese Medicine, Guiyang, 550025 China

**Keywords:** *Dipsacus asperoides*, Dipsacus saponin VI, Diversity, Endophytic fungi, Evolutionary system, Fermentation

## Abstract

**Abstract:**

*Dipsacus asperoides* contains multiple pharmacologically active compounds. The principal are saponins. The plant can be cultivated, but it contains lower levels of bioactive compounds than the plant in the wild. It may be the reason to exploit the endophytic fungi that colonize the plant roots in order to produce bioactive compounds. However, the endophytic fungi of *D. asperoides* have not been analyzed in detail. In this study, we isolated and identified 46 endophytic fungal strains from the taproots, lateral roots and leaves, and we used morphological and molecular biological methods to assign them into 15 genera: *Fusarium* sp., *Ceratobasidium* sp., *Chaetomium* sp., *Penicillium* sp., *Aspergillus* sp., *Talaromyces* sp., *Cladosporium* sp., *Bionectria* sp., *Mucor* sp., *Trichoderma* sp., *Myrothecium* sp., *Clonostachys* sp., *Ijuhya* sp., *Leptosphaeria* sp. and *Phoma* sp. Taproots contained abundant endophytic fungi, the numbers of which correlated positively with level of dipsacus saponin VI. Primary fermentation of several endophytic fungal strains from taproots showed that *Fusarium, Leptosphaeria, Ceratobasidium* sp. and *Phoma* sp. can produce the triterpenoid saponin. These results may guide efforts to sustainably produce bioactive compounds from *D. asperoides*.

**Graphical abstract:**

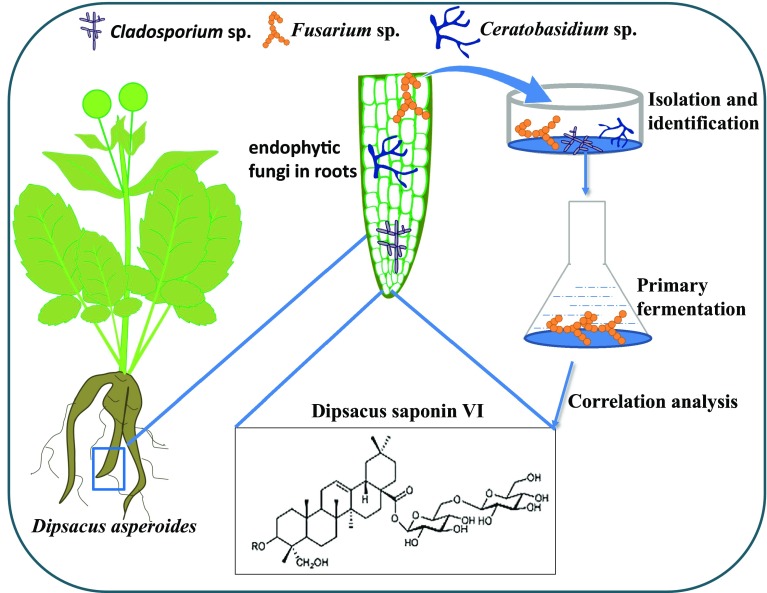

**Electronic supplementary material:**

The online version of this article (10.1007/s11274-019-2616-y) contains supplementary material, which is available to authorized users.

## Introduction

*Dipsacus asperoides* is a well-known medicinal plant used to curing occlusion diseases, punch injury, and rheumatism (Niu et al. 2015; Wong et al. 2007). Saponins, the major bioactive compound in *D. asperoides*, are isolated primarily from the taproots and widely used to treat fractures (Zhang et al. 2003; Jung et al. 2012). *D. asperoides* in the wild has diminished as a result of exploitation (Zhang et al. 1997; Chen et al. 2014; Wang et al. 2016), and the cultivated plant contains lower levels of dipsacus saponin VI than the plant in the wild. Therefore, rapid, efficient and environmentally sustainable methods are needed to obtain this and other saponins from *D. asperoides* (Cira et al. 2008; Jiao et al. 2015).

It may be possible to obtain saponins from the endophytic fungi that colonize *D. asperoides* (Jiao et al. 2015). Such fungi colonize the flowers, seeds, taproots, stems and leaves of many plant species, without causing visible disease symptoms (Aly et al. 2011). Endophytes establish a long-term symbiotic relationship with their plant hosts (Zuccaro et al. 2011). Some endophytic fungi and their metabolites increase resistance to plant pathogens and tolerance to drought (Redman et al. 2002; Waller et al. 2005; Herre et al. 2007; Rodriguez and Redman 2008). Endophytic fungi can combine with other endogenous microorganisms and antibacterial compounds to form a defense system that produces alkaloids to strengthen immunity and maintain growth under stress (Qin et al. 2011; Clay and Holah 1999). Some endophytic fungi produce active proteases helped maintain plant activities, such as pectinase and esterase, which degrade cell walls (Zhao et al. 2016). Pathology can result when programmed senescence in the plant or environmental change perturb the fungal population in the plant (Stamford et al. 2001).

In addition to supporting the growth and productivity of medicinal plants, endophytic fungi can produce bioactive metabolites similar to plant hosts, making them a potential source of medicinal compounds (Chandra 2012). For example, the endophytic fungus isolated from *Taxus chinensis* can be produced the anti-cancer compound paclitaxel (Li et al. 2009b). Other endophytic fungi produce the drug compounds camptothecin, podophyllotoxin (Eyberger et al. 2006; Puri et al. 2006), hypericin and emodin (Kusari et al. 2009). Endophytic fungi can produce bioactive compounds through industrial fermentation (Winter et al. 2011; Walsh and Fischbach 2010) (Kusari and Spiteller 2011). The research of endophytic fungi may provide new ideas and methods for developing bioactive compounds in medicinal plants in ways that sustain the development of traditional Chinese medicine resources (Zuccaro et al. 2011).

Endophytic fungi have been analyzed in at least 145 species of medicinal plants, but no such analysis has been reported for *D. asperoides*, to the best of our knowledge. Here we characterized the taxonomic diversity of *D. asperoides* taproots, lateral roots and leaves, and analyzed the correlation between the number of fungi and the level of saponins. Primary fermentation was performed with several endophytic fungal strains to examine the possibility of large-scale development of natural products.

## Materials and methods

### Sample collection

Two-year-old *D. asperoides* from Meihuashan in Weining County, Guizhou Province (N26°23′10.46′′) was planted at the Guiyang University of Chinese Medicine (E106°37′41.64′′). Plant material was washed and soaked in 0.1% SDS for 10 min, then rinsed with double-distilled water. The material was divided into taproots, lateral roots and leaves, which were stored at 4 °C.

### Isolation of endophytic fungi

The surface of plant material was sterilized by soaking in 0.1% mercuric chloride for 5 min, then in 75% ethanol for 3 min. The disinfected material was rinsed three times (1 min each time) with sterile water. The material was cut into pieces measuring 0.5 × 0.5 cm, and incubated at 28 °C on petri dishes containing potato dextrose agar (PDA), tryptone soy agar (TSA), beef extract tryptone agar (NA) and Luria–Bertani (LB) medium. Five biological replicates were prepared for each tissue, and growth was monitored every day. Endophytic fungal strains were inoculated on PDA slant culture-medium. After fungal cultures were fully grown, slant culture tubes were closed with tampons wrapped with oilpaper and stored at 4 °C.

### Taxonomic identification of endophytic fungi

Identification of endophytic fungi was accomplished following the methods described by Cannon et al. (Cannon and Simmons 2002). In this study, we perform its molecular reidentification, based on the analysis of internal transcript spacer (ITS) regions of endophytic fungi (Ding et al. 2018; Koljalg et al. 2005). Colonial morphology of endophytic fungi was identified using the point planting method as described (Chen et al. 2012). In brief, fungal spores were inoculated onto the center of solid PDA and incubated at 28 °C. Fungal characteristics were recorded every day, including colony shape, height and color of aerial hyphae, base color, growth rate, margin, surface texture, and depth of growth into the agar. At least three cultures were characterized on each petri dish, and on the attempts equated colony morphologies from different plates of the same plant. Endophytic fungi were preliminarily assigned to genera based on spore and culture characteristics.

The sequence analysis was also performed to assist in specimen identification. Mycelium was gathered directly from the surface of 4-day-old agar cultures and ground into a powder in liquid nitrogen. The powder was suspended in buffer [200 mM Tris–HCl (pH 8.0), 25 mM EDTA (pH 8.0), 250 mM NaCl and 0.5% SDS (pH 7.5)]. DNA was extracted using phenol and chloroform, and precipitated in ethanol. DNA integrity was analyzed by agarose gel electrophoresis, and purity was assessed using a Micronuclear Quantifier (Nanodrop 2000, Thermo Scientific, USA).

Internal transcript spacer (ITS) regions of endophytic fungi were amplified using polymerase chain reaction (PCR) and the universal ITS primers, V9D (5′-TTAAGTCCCTGCCCTTTGTA-3′) and LS266 (5′-GCATTCCCAAACAACTCGACTC-3′). Reactions (25 µL) contained 100 ng of genomic DNA, 10 µM of each primer, 12 µL of Premix Taq^™^ (Ex Taq™ 2.0 plus dye) and sterile double-distilled water. Thermal cycling parameters for PCR were as follows: pre-denaturation at 94 °C for 5 min; 30 cycles of denaturation at 94 °C for 30 s, annealing at 53 °C for 30 s and extension at 72 °C for 2 min; and a final extension step at 72 °C for 10 min. PCR products were detected on 1.2% (w/v) agarose gel prepared in 1× TAE buffer and electrophoresed at 100 V for 45 min.

Fragments were eluted and sent to be sequenced by Kingsley Biotech (Nanjing, China). Further information to guide the taxonomic identification of fungal strains came from the Flora of Chinese Mycology. BLAST searches of fungal sequences were conducted to analyze homology with identified sequences in ITS. Moreover, the comparison analysis of UNITE database to complement GenBank results. Tree topologies were evaluated using bootstrap analyses in MEGA6 (1000 bootstrap replicates). Phylogenetic trees were inferred using the neighbor-joining method.

### Analysis of endophytic fungal diversity

Menhinick’s index (*Dmn*) was used to quantify species richness among the isolated endophytic fungi. *Dmn* was calculated as $Dmn=S/\sqrt N$, where *S* refers to the number of different endophytic fungal species, and *N* refers to the total number of isolated endophytic fungi. The Shannon diversity index (*H*′) was calculated using $H'= - \sum\nolimits_{i}^{k} {Pi(LnPi)}$, where *Pi* = *Ni*/*N, Ni* is the number of individuals of the species, and *k* is the number of different endophytic species in a sample. The isolation rate (IR) was calculated by dividing the total number of isolates in a trial by the total number of samples in the trial. IR was used to measure the richness of endophytic fungi in plant tissues. The Sorensen similarity index (*Cs*) was calculated as *Cs* = 2*j*/(*a* + *b*), where *j* is the number of endophytic fungi common to the two tissues being compared, and *a* and *b* are the numbers of endophytic fungi in each tissue. *Cs* was used to quantify species similarity between different tissues.

### Quantification of dipsacus saponin VI

Samples of *D. asperoides* taproots, lateral roots and leaves were dried and ground into powder. Sample powder were soaked in methanol solution, ultrasonicated for 30 min (power, 100 W; frequency, 40 kHz), allowed to cool, weighed, and membrane-filtered. The filtered sample was analyzed for dipsacus saponin VI on a C18 symmetry column (4.6 × 250 mm, 5 µm) on a Waters HPLC system, with the following chromatography parameters: mobile phase, acetonitrile–water (30:70); flow rate, 1.0 mL/min; injection volume, 20 µL; detection wavelength, 212 nm; and theoretical plate number, ≥ 3000. HPLC run time was 25 min (Pharmacopoeia of the People’s Republic of China, 2015).

HPLC was also conducted with standard dipsacus saponin VI (purity, 91.3%; JY8R—BINA2), which was obtained from the China Food and Drug Certification Research Institute (Beijing, China). The standard was dissolved in methanol to a concentration of 0.15 mg/mL. Retention time of the standard was 18.254 min under our conditions.

### Fermentation of endophytic fungi

Taproot mycelium were transferred to an Erlenmeyer flask containing 100 mL liquid medium and cultured at 28 °C for 5 days with shaking at 160 rev min^−1^. Fungal characteristics were recorded every day, including color, viscosity, odor and other properties of the fermentation broth. Samples of mycelium (100 mg) were harvested by filtering and ground into powder in liquid nitrogen. DNA was extracted and PCR-amplified as described above (Cannon and Simmons 2002).

### Statistical analyses

All results were expressed as mean ± SEM. Graphs were prepared using GraphPad Prism 7.0. Differences between mean values were assessed for significance using one-way analysis of variance (ANOVA), followed by the least significant difference (LSD) test for *post hoc* comparisons (equal variances were assumed). Significance was indicated as follows: *P < 0.05, **P < 0.01, and ***P < 0.005.

## Results

### Identification of endophytic fungi from *D. asperoides* roots and leaves

**Fig. 1:**
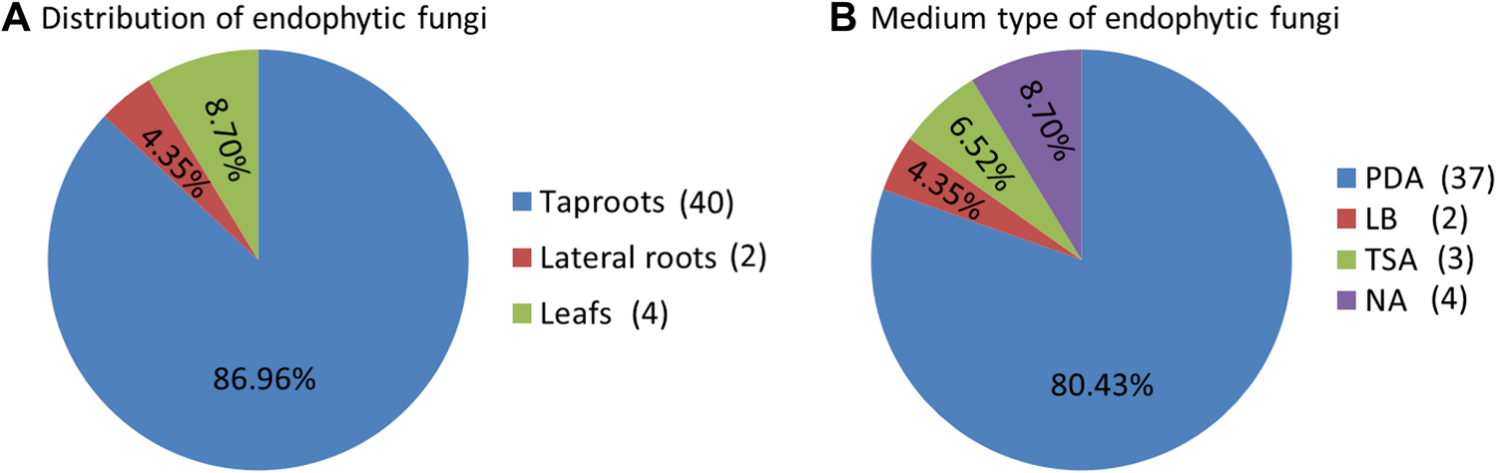
Isolation of endophytic fungi from *Dipsacus asperoides*. Tissues of *Dipsacus asperoides* were cultured in the culture medium of PDA, LB, TSA and NA. 46 isolates were identified in 100 taproot segments, 100 lateral root segments and 100 leaf segments based on their morphological characteristics. **A** Distribution of endophytes in different tissues of *D. asperoides*: 40 strains (86.96%) were isolated from the taproots, 2 strains (4.35%) were isolated from lateral roots and 4 strains (8.70%) were isolated from the leaves. **B** Culture of different endophytes from different *D. asperoides* tissues on different media: PDA supported growth of 37 strains (80.43%); LB medium, 2 strains (4.35%); TSA, 3 strains (6.52%); and NA, 4 strains (8.70%)

**Fig. 2:**
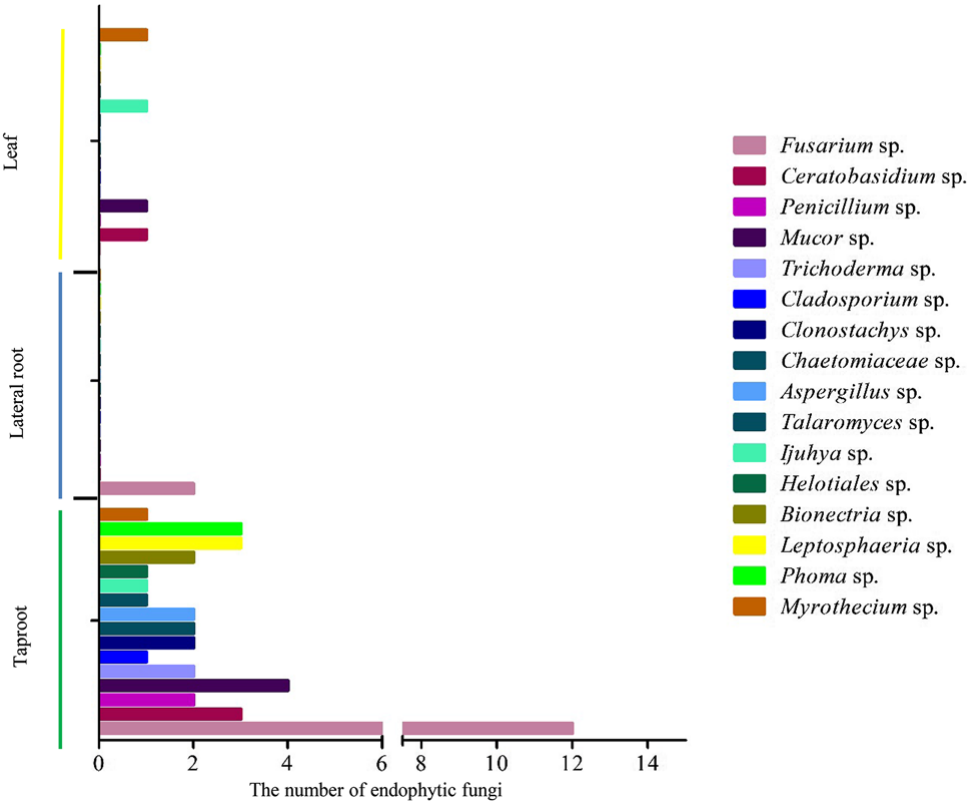
Diversity of endophytic fungi from *D. asperoides*. Statistical histogram of the number of different endophytic fungi in the taproots (green), lateral roots (blue) and leaves (orange)

Different tissues of *D. asperoides* were cultured in PDA, LB, TSA and NA culture media. A total of 46 strains were isolated and preliminarily assigned based on colony and hyphal characteristics (Fig. 1). The largest number of endophytic fungal isolates (40) were found in taproots, followed by leaves (4) and lateral roots (2) (Fig. 1A). The isolates in four media showed that the greatest number was obtained in PDA (37), followed by NA (4), TSA (3) and finally LB medium (2) (Fig. 1B). The IR in taproots (0.40) was significantly higher than that in lateral roots (0.02) or leaves (0.04). Taproots also showed that *H*′ and *Dmn* were higher than leaves and lateral roots (Table 1; Fig. 2). These results suggest that the taproots may provide the best niche or entry point for colonization and penetration by endophytic fungi.

**Table 1:** Colonisation, isolation, species richness and multiple infection rates of endophytic fungi at each healthy tissue of *Dipsacus asperoides*

Parameter	Taproots	Lateral roots	Leaves	Total
No. of samples	100	100	100	300
Isolation rate (IR)	0.40	0.02	0.04	0.46
Shannon diversity index (*H*′)	2.60	0.00	1.40	4.00
Menhinick’s index (*Dmn*)	2.53	0.71	2.00	5.24

Diversity statistical table of endophytic fungi in *D. asperoides* taproots, lateral roots and leaves. Indicated are the number of isolates recovered, isolation rate (IR), Shannon diversity index (*H*′), and Menhinick’s index (*Dmn*)

**Fig. 3:**
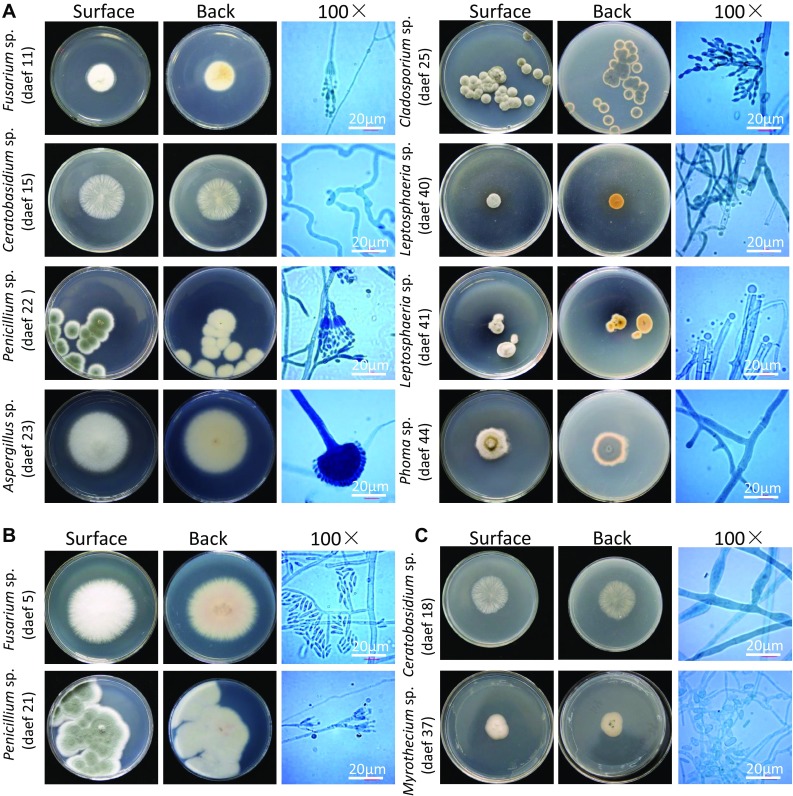
Morphological characteristics of endophyte fungi. Photographs showing typical morphology of endophyte fungi from taproots, lateral roots and leaves of *D. asperoides*. **A** Characteristics of endophytic fungi isolated from the taproots, showing “surface”, “back” and microstructure. These characteristics were observed for the following isolates: daef 11, 15, 22, 23, 25, 40, 41 and 44. Scale bar, 20 µm. **B** Characteristics of endophytic fungi isolated from the lateral roots, showing “surface”, “back” and microstructure. These characteristics were observed for daef 5 and 21. Scale bar, 20 µm. **C** Characteristics of endophytic fungi isolated from the leaves, showing “surface”, “back” and microstructure. These characteristics were observed for daef 18 and 37. Scale bar, 20 µm

Microscopic analysis of the 46 endophytic fungi allowed them to be assigned preliminarily to *Fusarium* sp. (samples daef 1–14) on the basis of their irregular, round shape and hyphae uplift, fast growth, yellow pigment production, and presence of conidia or spores; to *Ceratobasidium* sp. (daef 15–18) on their basis of their loose white hyphae and lack of conidia; to *Chaetomiaceae* sp. (daef 19–20) on the basis of white colonies and soft hair or cotton with yellow pigment on the back of hyphae; to *Penicillium* sp. (daef 21–22) on the basis of scattered hyphae, pigment production, and broom-like stem with a string of conidia; to *Aspergillus* sp. (daef 23) on the basis of white, pilose, cotton-like hyphae with erect hyphae, conidiophores and a hemispherical capsule; to *Talaromyces* sp. (daef 24) on the basis of green cotton-like hyphae and a small, broom-like stem with conidiophores; to *Cladosporium* sp. (daef 25) on the basis of green villi-like hyphae with small water-like substances, elliptical and round conidia; and to *Bionectria* sp. (daef 26–27) on the basis of white cotton-like appearance with some water-like substances, broom-like branches with long spindle-shaped spore stalks on the branchlets, and small curved elliptical and ovate spores on the spore stalks (Figs. 3 and S1).

The remaining endophytic fungal isolates were assigned to genera based on comparison with known fungi: *Clonostachys* sp. (daef 28–29), *Mucor* sp. (daef 30–34), *Trichoderma* sp. (daef 35–36), *Myrothecium* sp. (daef 37), *Ijuhya* sp. (deaf 38–39), *Leptosphaeria* sp. (daef 40–42), *Phoma* sp. (daef 43–45) and *Heliogales* sp. (daef 46).

Comparison of ITS sequences from the 46 isolates with fungal sequences in GenBank (Table 2) lead to the identification of 15 genera: *Fusarium* sp., *Ceratobasidium* sp., *Chaetomium* sp., *Penicillium* sp., *Aspergillus* sp., *Talaromyces* sp., *Cladosporium* sp., *Bionectria* sp., *Mucor* sp., *Trichoderma* sp., *Myrothecium* sp., *Clonostachys* sp., *Ijuhya* sp., *Leptosphaeria* sp. and *Phoma* sp. Two strains that could not be assigned to a genus were identified as *Chaetomiaceae* sp. and *Helotiales* sp. based on GenBank analysis. Taxonomic identification based on ITS sequencing was consistent with that based on morphological observation. In addition, the results of blastn analysis by UNITE database were consistent with NCBI analysis (Table S1).

**Table 2:** Similarity between the isolates and closest species in GenBank

Strain ID	Accession no.	Closest (Accession no.)	Similarity (%)
daef1	MH550471	*Fusarium oxysporum* (KU872828.1)	99
daef2	MH550472	*Fusarium globosum* (LT746280.1)	99
daef3	MH550473	*Fusarium solani* (KY484984.2)	98
daef4	MH550474	*Fusarium* sp. (JF740911.1)	99
daef5	MH550475	*Fusarium tricinctum* (MG274296.1)	99
daef6	MH550476	*Fusarium lateritium* (AF310980.1)	99
daef7	MH550477	*Fusarium acuminatum* (KJ082098.1)	99
daef8	MH550478	*Fusarium* sp. (LT746244.1)	98
daef9	MH550479	*Fusarium* sp. (AF310976.1)	98
daef10	MH550480	*Fusarium acuminatum* (HM068320.1)	98
daef11	MH550481	*Fusarium lateritium* (AF310980.1)	99
daef12	MH550482	*Fusarium* sp. (LT746240.1)	99
daef13	MH550483	*Fusarium* sp. (LT746244.1)	99
daef14	MH550484	*Fusarium proliferatum* (LT841264.1)	99
daef15	MH550485	*Ceratobasidium* sp. (DQ520098.1)	97
daef16	MH550486	*Ceratobasidium* sp. (KC782938.1)	99
daef17	MH550487	*Ceratobasidium* sp.(DQ097889.1)	96
daef18	MH550488	*Ceratobasidium* sp.(AF354091.1)	99
daef19	MH550489	*Chaetomiaceae* sp. (KC007192.1)	99
daef20	MH550490	*Chaetomium megalocarpum* (KC109744.1)	99
		*Chaetomium pseudocochliodes* (JN209925.1)	98
daef21	MH550491	*Penicillium janthinellum* (MG938669.1)	98
daef22	MH550492	*Penicillium* sp. (KX961210.1)	98
		*Penicillium skrjabinii* (EU427287.1)	99
daef23	MH550493	*Aspergillus lentulus* (KX903293.1)	99
		*Aspergillus viridinutans* (EF661280.1)	99
daef24	MH550494	*Talaromyces apiculatus* (JN899375.1)	98
daef25	MH550495	*Cladosporium cladosporioides* (KP701868.1)	99
		*Cladosporium pseudocladosporioides* (KP701943.1)	99
		*Cladosporium delicatulum* (KP701939.1)	98
daef26	MH550496	*Bionectria* sp. (KF367470.1)	99
daef27	MH550497	*Bionectria* sp.(KF367477.1)	99
daef28	MH550498	*Clonostachys rosea f*. (HM751081.1)	99
daef29	MH550499	*Clonostachys* sp. (KC806284.1)	99
		*Clonostachys pseudochroleuca* (KC806259.1)	99
daef30	MH550500	*Mucor racemosus* (KJ911228.1)	99
daef31	MH550501	*Mucor* sp. (KU060772.1)	98
daef32	MH550502	*Mucor fragilis* (JQ972062.1)	97
daef33	MH550503	*Mucor circinelloides f*. (JN205987.1)	96
daef34	MH550504	*Mucor fragilis* (JQ972063.1)	97
daef35	MH550505	*Trichoderma hamatum* (KM491888.1)	99
daef36	MH550506	*Trichoderma asperellum* (KF723005.1)	99
		*Trichoderma koningiopsis* (GQ229070.1)	99
daef37	MH550507	*Myrothecium roridum* (FJ914699.1)	99
		*Myrothecium* sp. (KY086248.1)	99
		*Myrothecium verrucaria* (KM215639.1)	97
daef38	MH550508	*Ijuhya corynospora* (KY607539.1)	96
daef39	MH550509	*Ijuhya vitellina* (KY607531.1)	95
daef 40	MH550510	*Leptosphaeria* sp. (KJ934197.1)	99
daef41	MH550511	*Leptosphaeria* sp. (AJ317958.1)	99
daef42	MH550512	*Leptosphaeria biglobosa* (KY221834.1)	99
daef43	MH550513	*Phoma exigua* var. (EU343130.1)	99
daef44	MH550514	*Phoma exigua* var. (EU343168.1)	98
daef45	MH550515	*Phoma exigua* var.(EU343118.1)	98
daef46	MH550516	*Helotiales* sp. (FN548161.1)	99
		*Helotiales* sp. (MG066445.1)	99

Fungi were grouped into OTUs defined by 97% internal transcribed spacer (ITS) sequence similarity

The statistical table shows the similarity of the rDNA-ITS sequence of endophytic fungi from *D. asperoides* to the closest fungal sequences in GenBank, based on BLAST alignment. The strain ID has the format: Latin initials of *Dipsacus asperoides*, the initial letter of the endophytic fungus and the strain number. The GenBank accession number is also shown, with “Closest (Accession No.)” indicating the most similar fungus (and its accession number) from GenBank. Similarity (%) is the Ident value obtained by comparing the sequences between the two strains

The two dominant genera were *Fusarium* sp. to which 29.09% of isolates, and *Ceratobasidium* sp. to which 10.91% of isolates. *Myrothecium* sp. was isolated only from leaves. *Cs* analysis showed that the tissue pair with greatest similarity was lateral roots and leaves (*Cs* 2.00), followed by taproots and lateral roots (1.88) and finally taproots and leaves (1.79). These results suggest the heterogeneity of the endophyte assemblage.

**Fig. 4:**
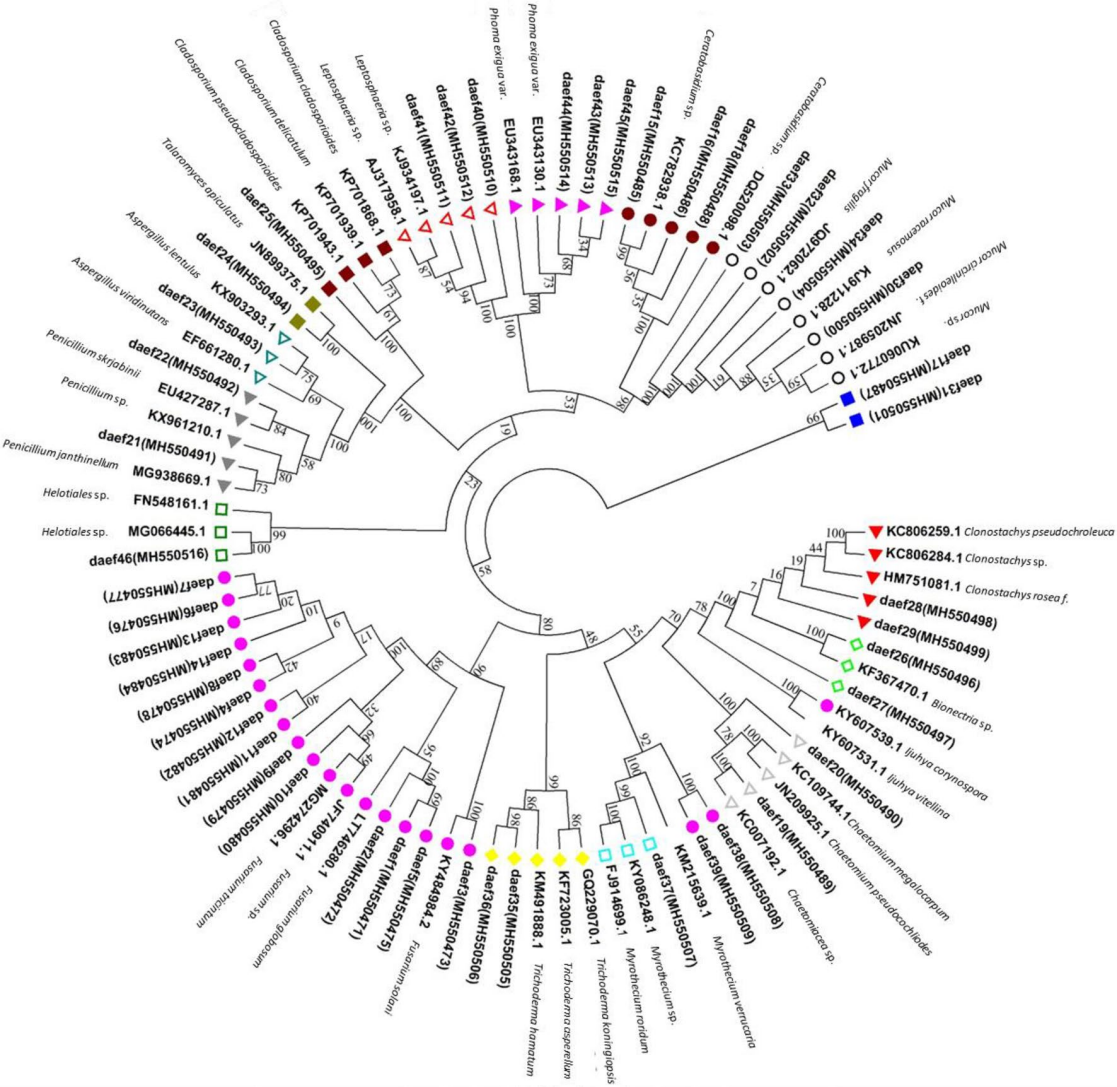
Phylogenetic identification of endophytic fungi from *D. asperoides*. Phylogenetic tree based on neighbor-joining analysis of ITS sequences from the 46 strains of endophytic fungi isolated from taproots, lateral roots and leaves. ITS sequences obtained were submitted to the NCBI database, and BLAST searches were performed to select species showing 95–100% homology with the isolated species. Closely related species are labeled with taxonomic names, followed by the accession number. Significant bootstrap values are indicated at the branching points

A phylogenetic tree based on ITS sequences (Fig. 4) assigned *Fusarium* sp. isolates to six clusters, three of which were closely related and clustered with *Fusarium globosum* (LT746280.1), two of which clustered with *Fusarium tricinctum* (MG274296.1) and *Fusarium* sp. (JF740911.1), and one of which was related to *Fusarium solani* (KY484984.2). Clades comprised daef 6, 7, 13 and 14; daef 4 and 8; and daef 11 and 12. *Fusarium* sp. was the most frequently isolated fungal genus. The four isolates daef 30 and 32–34 were grouped into a branch with the reference taxon *Mucor* sp. The daef 15, 16 and 18 and *Ceratobasidium* sp. were grouped into a branch with 100% bootstrap support, with daef 15 clustering with *Ceratobasidium* sp. (KC782938.1).

*Leptosphaeria* isolates formed a cluster with reference taxa *Leptosphaeria* sp. (KJ934197.1 and AJ317958.1). *Phoma* sp. isolates formed a cluster with reference taxa *Phoma exigua* var. (EU343130.1 and EU343168.1). *Penicillium* sp. isolates were grouped into two clusters: daef 21 clustered with *Penicillium janthinellum* (MG938669.1), and daef 22 clustered with *Penicillium skrjabinii* (EU427287.1). *Trichoderma* isolates formed a cluster with *Trichoderma hamatum* (KM491888.1), *Trichoderma asperellum* (KF723005.1) and *Trichoderma koningiopsis* (GQ229070.1). *Clonostachys* sp. isolates formed a cluster with *Clonostachys rosea* f. (HM751081.1), *Clonostachys* sp. (KC806284.1) and *Clonostachys pseudochroleuca* (KC806259.1). A *Bionectria* sp. isolate formed a cluster with *Bionectria* sp. (KF367470.1) with 100% bootstrap support. *Myrothecium* sp. isolates were grouped into two clusters: two closely related isolates formed a clade and one isolate formed a clade with *Myrothecium roridum* (FJ914699.1) and *Myrothecium* sp. (KY086248.1) with 99% bootstrap support. *Chaetomium* sp. isolates formed a cluster with *Chaetomium* sp. (KC007192.1), *Chaetomium pseudocochliodes* (JN209925.1) and *Chaetomium megallocarpum* (KC109744.1). The *Talaromyces* sp. isolate formed a cluster with *Talaromyces apiculatus* (JN899375.1) with 100% bootstrap support. The *Aspergillus* sp. isolate formed a cluster with *Aspergillus lentulus* (KX903293.1) and *Aspergillus viridinutan*s (EF661280.1). The *Cladosporium* sp. isolate formed a cluster with *Cladosporium pseudocladosporioides (KP701943.1), Cladosporium delicatulum (KP701939.1)* and *Cladosporium cladosporioides (KP701868.1)* with 100% bootstrap support. The daef 17 and 31 could not be represented in the phylogenetic tree because of low sequence quality. The daef 46 clustered with *Helotiales* sp. (FN548161.1 and MG066445.1) with 99% and 100% bootstrap support.

### Positive correlation between dipsacus saponin VI level and number of endophytic fungi in roots

**Fig. 5:**
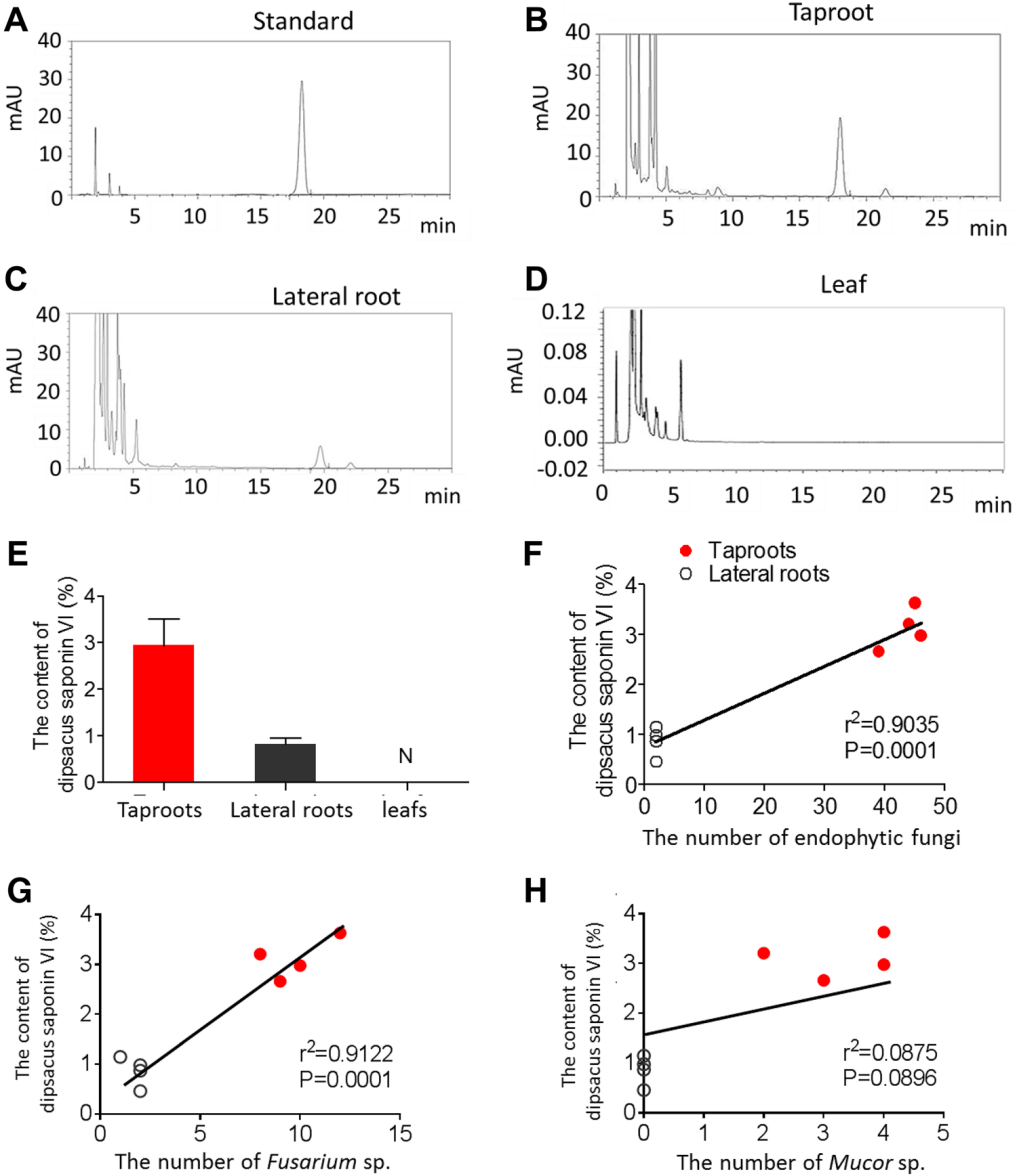
Dipsacus saponin VI level positively correlated with endophytic fungi in roots of *D. asperoides*. Dipsacus saponin VI was quantified in taproots, lateral roots and leaves using HPLC. **A** Chromatogram of the standard dipsacus saponin VI. The y-axis indicates the absorbance of dipsacus saponin VI, and the x-axis indicates the measurement time (min). **B** Chromatogram of dipsacus saponin VI in taproots. **C** Chromatogram of dipsacus saponin VI in lateral roots. **D** Chromatogram of dipsacus saponin VI in leaves. **E** Quantification of dipsacus saponin VI content in different tissues. Data are mean ± SEM (n = 4). ***P < 0.005 (one-way ANOVA and least significant difference test *post hoc*). **F** Correlation analysis between dipsacus saponin VI level and the number of endophytic fungi in taproots and lateral roots. Each isolate is represented by a spot (n = 4, R^2^ = 0.9035, P = 0.0001). **G** Correlation analysis between dipsacus saponin VI level and the number of *Fusarium* sp. in taproots and lateral roots. Each isolate was represented as a spot (n = 4, R^2^ = 0.9122, P = 0.0001). **H** Correlation analysis between the dipsacus saponin VI level and the number of *Mucor* sp. in taproots and lateral roots. Each isolate was represented as a spot (n = 4, R^2^ = 0.0875, P = 0.0896)

Dipsacus saponin VI was quantified in taproots, lateral roots and leaves using HPLC (Fig. 5A–D). Levels differed significantly in different tissues (P < 0.05). Levels were highest in taproots (2.98%), lower in lateral roots (0.87%) and below the detection limit in leaves (Fig. 5E). Level of dipsacus saponin VI positively correlated with the total number of endophytic fungi (Fig. 5F) and with the number of *Fusarium* sp. (Fig. 5G), but independent with the number of *Mucor* sp. in taproots (Fig. 5H).

### Primary fermentation of endophytic fungi

**Fig. 6:**
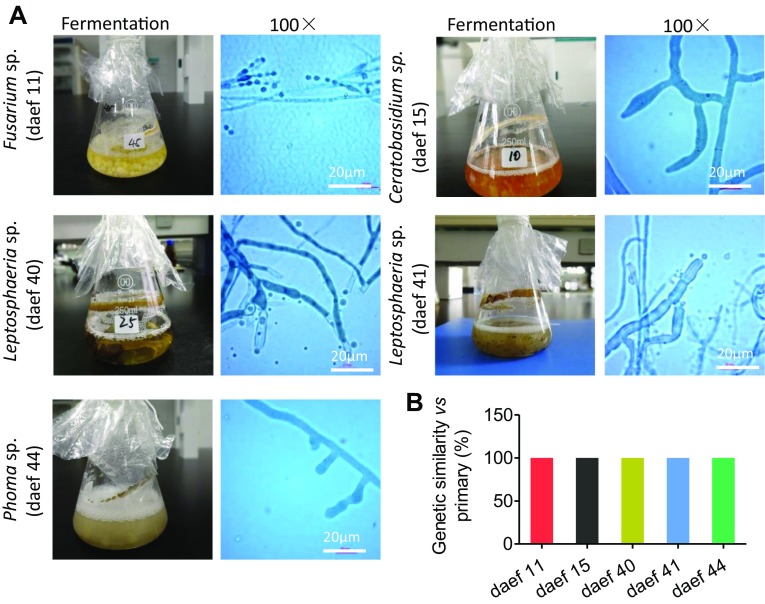
Primary fermentation of endophytic fungi from *Dipsacus asperoides*. Five endophytic fungi that were enriched in taproots and came from different genera were subjected to primary fermentation: daef 11 (*Fusarium* sp.), 40 and 41 (*Leptosphaeria* sp.), 15 (*Ceratobasidium* sp.) and 44 (*Phoma* sp.). **A** Photographs of the fermentation of five strains of endophytic fungi and their microscopic morphology. Scale bar, 20 µm. **B** Quantification of synergistic alignment between ITS sequences of the foaming fungus (ITS’) and the ITS sequences of the original fungal isolate

Selected endophytic fungal isolates were subjected to primary fermentation tests to identify which strains may produce dipsacus saponin VI. Several endophytic fungi enriched in taproots and from different genera were tested: daef 11 (*Fusarium* sp.), 40 and 41 (*Leptosphaeria* sp.), 15 (*Ceratobasidium* sp.) and 44 (*Phoma* sp.). Within 10 min at 60 °C, all these strains produced foam and showed no fading (Fig. 6A). These strains may produce saponins. In addition, daef 15 produced red pigment, while daef 40, 41 and 44 produced green or deep green pigments.

To verify that these fermented fungi were identical to the strains originally isolated and were not contaminated by other microorganisms, we confirmed that the microstructure of the fermented fungi was consistent with that of the original strains. We also amplified ITS regions from mycelium of the fermented strains and confirmed that the sequences were 100% homologous to the regions sequenced from the original strains (Fig. 6B and S2).

## Discussion

This study begins the process of correlating production of perhaps the most relevant bioactive compound from this plant, saponins, with the number and diversity of endophytic fungi in different tissues. Our results help clarify the biodiversity and phylogenetic relationships of endophytic fungi in *D. asperoides*, which can begin to shed light on how endophytic fungi can affect the quality of traditional Chinese medicinal plants.

The 46 endophytic fungi were isolated from different tissues of *D. asperoides*. This number is slightly lower than what has been reported with other plants, which may mean that some strains stopped colonizing *D. asperoides* over time, such as due to inhibition by other rapidly growing strains (Gonzaga et al. 2015).

Nearly all the fungal isolates in our study were colonized in taproots, while only two strains were isolated from lateral roots and four strains were isolated from leaves. This suggests that in this medicinal plant, the taproots are most likely to be colonized. The much greater abundance of fungi in taproots reflects that fungi can penetrate host plants via the roots, where they gain access to nutrients in xylem and phloem (Martin et al. 2012; Pfurtscheller and Klimesch 1990; Sheng-Liang et al. 2014). The diversity of endophytic fungi in taproots was more higher than that in other tissues. We guess that these fungi colonized *D. asperoides* as spores that moved from the soil to the roots; the lateral roots acted simply as transport bridges to carry the fungi to taproots for storage (Courty et al. 2018). In contrast to our results, diversity of endophytic fungi was greatest in the leaves of *Gossypium hirsutum* (Li et al. 2014) and *Miscanthus* × *giganteus* (Schmidt et al. 2018).

Endophytic fungi in plants are primarily Ascomycetes and their anamorphs, although they can also be Basidiomycetes, Zygomycetes, and Oomycetes (Soca-Chafre et al. 2011). In *D. asperoides*, endophytic fungi included many rare species, mainly belong to Deuteromycota, that accounted for 48.94% of isolates; Ascomycetes accounted for only 34.04% of isolates. Some Basidiomycetes and Zygomycetes were observed.

*Fusarium* sp. is the predominant microflora in *D. asperoides*. This genus occurs as an endophyte in various cash crops, including *Solanum lycopersicum* (Aime et al. 2013), *Drepanocarpus lunatus* (Liu et al. 2016), and *Dioscorea zingiberensis* (Zhang et al. 2009). *Ceratobasidium* sp. can cause sheath blight and act as a saprotroph in rice (Mosquera-Espinosa et al. 2013), persimmon (Ceresini et al. 2012) and soybean (Salehi et al. 2005). *Aspergillus* sp. acts as an endophyte of *Opuntia dillenii* and several other plants (Li et al. 2009a). *Myrothecium* sp. acts as an endophyte of *Calophyllum apetalum* and *Garcinia Morella* (Ruma et al. 2015).

Several of the endophytic fungi that were identified in *D. asperoides* can produce bioactive compounds of medicinal interest. *Trichoderma* sp., *Talaromyces* sp., *Mucor* sp. and *Penicillium* sp. can produce proteases that degrade cellulose (Zhao et al. 2016; Thongekkaew et al. 2013), dairy products, and polysaccharides (Inoue et al. 2015). *Fusarium* sp. can produce triterpenoid saponins (Cira et al. 2008; Jiao et al. 2015), which are the main secondary metabolites of *D. asperoides* and used to treat osteoporosis, reduce lipids and protect against oxidation (Wang et al. 2016). Our results suggest that the main location of saponin production in *D. asperoides* is roots. Levels of dipsacus saponin VI were higher in taproot than in other tissues, and the taproot have been also the greatest number of endophytic fungi. In contrast, dipsacus saponin VI levels in leaves were below the limit of detection, perhaps due to scarcity of interactions between endophytic fungi and host, reflected in the relatively low *Dmn* and IR. Endophytes can prefer different plant tissues, where they form specific symbiotic relationships; as a result, different tissues contain different profiles of secondary metabolites (Jasinska et al. 2018; Jarvis et al. 1985; Liu et al. 2006; Wang et al. 2016).

Changing the environmental conditions of endophytes can lead them to produce different secondary metabolites (Eaton et al. 2010; Wang et al. 2017), increasing their usefulness as bioactive molecule factories. Many active pharmaceutical compounds have been isolated from filtrates of *Fusarium* sp. cultures (Suzuki et al. 2013). The leptosins I and J have been isolated from *Leptosphaeria* mycelium (Takahashi et al. 1994). A cyclic lipodepsipeptide has been isolated from *Phoma* sp. (Herath et al. 2009). When we subjected *Fusarium* sp., *Leptosphaeria* sp., *Ceratobasidium* sp. and *Phoma* sp. to primary fermentation, we found that all them could produce triterpenoid saponin. In addition, our isolates (*Cladosporium* sp., *Phoma* sp., *Fusarium* sp., and *Penicillium* sp.) were produced pigments that may be useful in the food, cosmetic and pharmaceutical industries. These results with primary fermentation may facilitate the development of strategies to produce natural products from *D. asperoides* (Bick and Rhee 1966; Zheng et al. 2017; Shah et al. 2015).

Our results highlight the diversity of endophytic fungi in medicinal plants and their ability to synthesize bioactive secondary metabolites (Gupta et al. 2018). They may also guide new approaches to synthesize dipsacus saponin VI from *D. asperoides*, permitting sustainable development of this important traditional Chinese medicine resource.

## Electronic supplementary material

Below is the link to the electronic supplementary material.

**Fig. S1**. Morphological characteristics of endophyte fungi. Photographs showing typical morphology of residual endophyte fungi in Fig. S1. Characteristics of endophytic fungi from *D. asperoides* taproots are shown at “surface” and “back”; microstructure is also shown. Scale bar, 20 μm. (PPTX 4076 KB)

**Fig. S2**. Homology comparison of endophytic fungi with foaming properties. Original sequence map of homology alignment between ITS sequences from the foaming fungi (ITS’) and the ITS sequences from the original fungal isolates, showing 100% homology for all the isolates (daef 11, 15, 40, 41 and 44). The alignment was produced using BioEdit 7.1.3. (PPTX 2070 KB)

**Table S1**. Similarity between the isolates from Dipsacus asperoides and the closest species in UNITE database. The statistical table shows the homology of the rDNA-ITS sequence of isolates from *D. asperoides* to the closest fungal sequences in UNITE database, based on blastn analysis. The strain ID has the format: latin initials of *Dipsacus asperoides*, the initial letter of the endophytic fungus and the strain number. The “Accession No. “is the GenBank accession number. The “Sequences producing significant alignments (Accession No.)” indicating the analysis of sequences between the fungi isolated from *Dipsacus asperoides* and 1 to 3 fungi from 15 best matches (as judged by the e value) fungi obtained from UNITE blastn comparative analysis. Identities (%) is the homology (similarity) obtained by comparing the sequences between the two strains. Score (Bits) is a log-scaled version of a score. It is a rescaled version of the raw alignment score that is independent of the size of the search space. The “E-value” is correction of the p-value for multiple testing. It indicates the possibility that the similarity between other sequences and the target sequence. The lower the score, the better. (DOCX 27 KB)
