# Supplementary material for: Association between dipsacus saponin VI level and diversity of endophytic fungi in roots of Dipsacus asperoides
Source: World J Microbiol Biotechnol. 2019 Feb 18;35(3):42. doi: 10.1007/s11274-019-2616-y (PMC6394449; doi:10.1007/s11274-019-2616-y)
Supplement: Supplementary file 1 — Fig. S1. Morphological characteristics of endophyte fungi. Photographs showing typical morphology of residual endophyte fungi in Fig. S1. Characteristics of endophytic fungi from D. asperoides taproots are shown at “surface” and “back”; microstructure is also shown. Scale bar, 20 μm. (PPTX 4076 KB) [file 11274_2019_2616_MOESM1_ESM.pptx]

## Slide 1
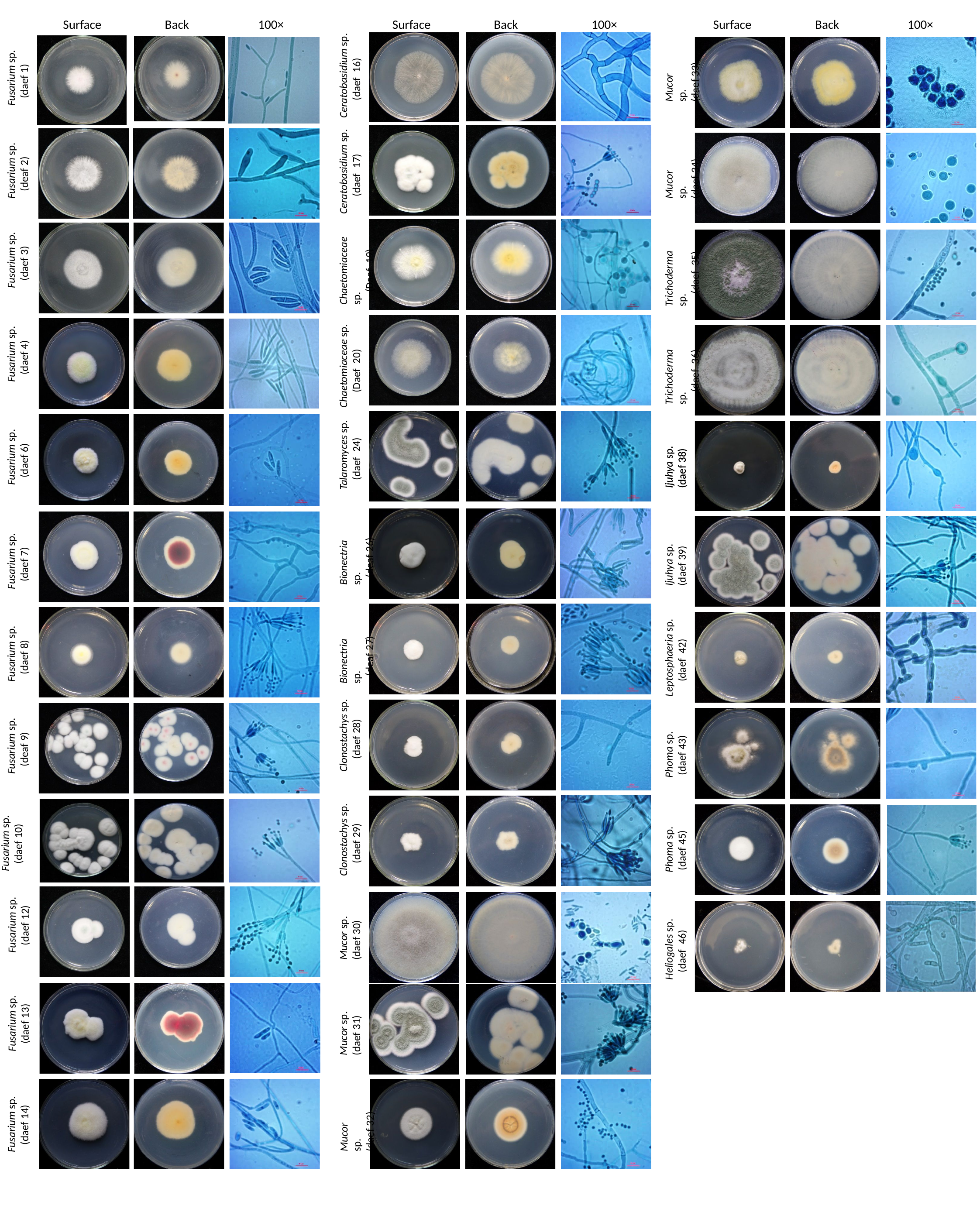

Surface
Back
100×
Surface
Back
100×
Surface
Back
100×
Ceratobasidium sp.
 (daef 16)
Fusarium sp.
 (daef 1)
Mucor sp.
(daef 33)
Fusarium sp.
 (deaf 2)
Ceratobasidium sp.
 (daef 17)
Mucor sp.
(daef 34)
Fusarium sp.
 (daef 3)
Chaetomiaceae sp.
 (Daef 19)
Trichoderma sp.
 (daef 35)
Fusarium sp.
 (daef 4)
Chaetomiaceae sp.
 (Daef 20)
Trichoderma sp.
 (daef 36)
Talaromyces sp.
 (daef 24)
Fusarium sp.
 (daef 6)
Ijuhya sp.
(daef 38)
Ijuhya sp.
(daef 38)
Bionectria sp.
 (deaf 26)
Fusarium sp.
 (daef 7)
Ijuhya sp.
(daef 39)
Fusarium sp.
 (daef 8)
Bionectria sp.
 (deaf 27)
Leptosphaeria sp.
 (daef 42)
Clonostachys sp.
 (daef 28)
Fusarium sp.
 (deaf 9)
Phoma sp.
 (daef 43)
Clonostachys sp.
 (daef 29)
Fusarium sp.
 (daef 10)
Phoma sp.
 (daef 45)
Fusarium sp.
 (daef 12)
Mucor sp.
(daef 30)
Heliogales sp.
 (daef 46)
20μm
Fusarium sp.
 (daef 13)
Mucor sp.
(daef 31)
Fusarium sp.
 (daef 14)
Mucor sp.
(daef 32)
