# Supplementary figures and images for: Association between dipsacus saponin VI level and diversity of endophytic fungi in roots of Dipsacus asperoides
Source: World J Microbiol Biotechnol. 2019 Feb 18;35(3):42. doi: 10.1007/s11274-019-2616-y (PMC6394449; doi:10.1007/s11274-019-2616-y)

## Slide 1
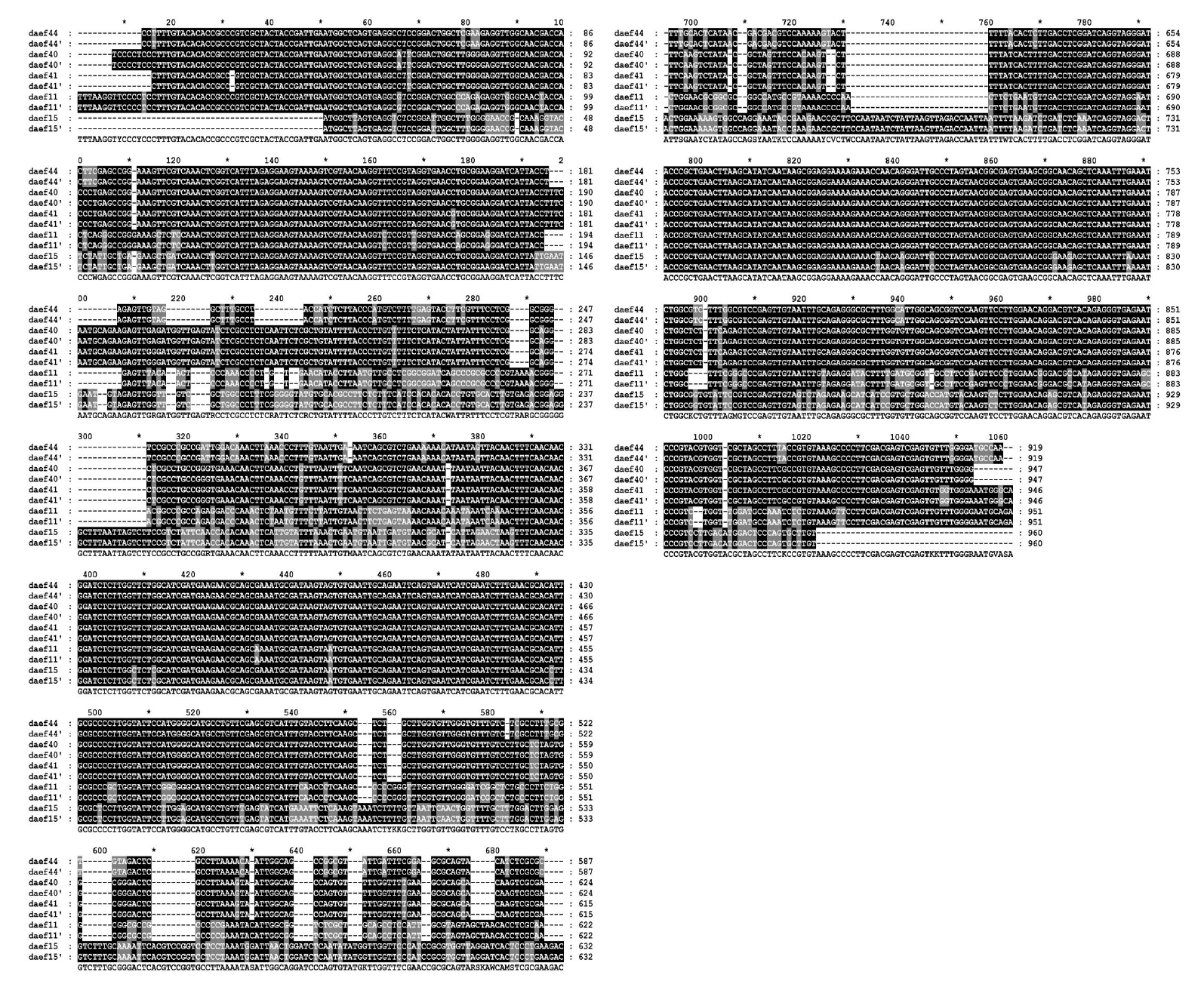

Supplement: Supplementary file 2 — Fig. S2. Homology comparison of endophytic fungi with foaming properties. Original sequence map of homology alignment between ITS sequences from the foaming fungi (ITS’) and the ITS sequences from the original fungal isolates, showing 100% homology for all the isolates (daef 11, 15, 40, 41 and 44). The alignment was produced using BioEdit 7.1.3. (PPTX 2070 KB) [file 11274_2019_2616_MOESM2_ESM.pptx]
