# Supplementary material for: Association between dipsacus saponin VI level and diversity of endophytic fungi in roots of Dipsacus asperoides
Source: World J Microbiol Biotechnol. 2019 Feb 18;35(3):42. doi: 10.1007/s11274-019-2616-y (PMC6394449; doi:10.1007/s11274-019-2616-y)
Supplement: Supplementary file 3 — Table S1. Similarity between the isolates from Dipsacus asperoides and the closest species in UNITE database. The statistical table shows the homology of the rDNA-ITS sequence of isolates from D. asperoides to the closest fungal sequences in UNITE database, based on blastn analysis. The strain ID has the format: latin initials of Dipsacus asperoides, the initial letter of the endophytic fungus and the strain number. The “Accession No. “is the GenBank accession number. The “Sequences producing significant alignments (Accession No.)” indicating the analysis of sequences between the fungi isolated from Dipsacus asperoides and 1 to 3 fungi from 15 best matches (as judged by the e value) fungi obtained from UNITE blastn comparative analysis. Identities (%) is the homology (similarity) obtained by comparing the sequences between the two strains. Score (Bits) is a log-scaled version of a score. It is a rescaled version of the raw alignment score that is independent of the size of the search space. The “E-value” is correction of the p-value for multiple testing. It indicates the possibility that the similarity between other sequences and the target sequence. The lower the score, the better. (DOCX 27 KB) [file 11274_2019_2616_MOESM3_ESM.docx]

Table S1 Similarity between the isolates and closest species in UNITE database

| Strain ID | Accession No. | Sequences producing significant alignments (Accession No.) | Identities (%) | Score  (Bits) | E-Value |
| --- | --- | --- | --- | --- | --- |
| daef1 | MH550471 | *Fusarium* ([AF310976](https://unite.ut.ee/bl_forw.php?id=108135)) | 96 | 1490 | 0 |
| daef2 | MH550472 | *Fusarium oxysporum* ([KC119203](https://unite.ut.ee/bl_forw.php?id=372189)) | 99 | 1494 | 0 |
| daef3 | MH550473 | *Fusarium* (EU750687) | 100 | 1599 | 0 |
|  |  | *Fusarium solani* (KU872821) | 99 | 1528 | 0 |
| daef4 | MH550474 | *Fusarium* ([EU552132](https://unite.ut.ee/bl_forw.php?id=43319)) | 99 | 1559 | 0 |
| daef5 | MH550475 | *Fusarium oxysporum* ([KU872849](https://unite.ut.ee/bl_forw.php?id=599561)) | 98 | 1510 | 0 |
|  |  | *Fusarium* ([KT282392](https://unite.ut.ee/bl_forw.php?id=549798)) | 99 | 1501 | 0 |
| daef6 | MH550476 | *Fusarium* ([GQ866861](https://unite.ut.ee/bl_forw.php?id=7200)) | 99 | 1557 | 0 |
| daef7 | MH550477 | *Fusarium* ([EU552132](https://unite.ut.ee/bl_forw.php?id=43319)) | 98 | 1546 | 0 |
| daef8 | MH550478 | *Fusarium* ([GU797143](https://unite.ut.ee/bl_forw.php?id=149818)) | 99 | 1555 | 0 |
| daef9 | MH550479 | *Fusarium* ([EU552132](https://unite.ut.ee/bl_forw.php?id=43319)) | 97 | 1487 | 0 |
| daef10 | MH550480 | *Fusarium* ([GU797143](https://unite.ut.ee/bl_forw.php?id=149818)) | 97 | 1490 | 0 |
| daef11 | MH550481 | *Fusarium* ([GQ866861](https://unite.ut.ee/bl_forw.php?id=7200)) | 99 | 1575 | 0 |
| daef12 | MH550482 | *Fusarium* ([AF310977](https://unite.ut.ee/bl_forw.php?id=108134)) | 96 | 1501 | 0 |
| daef13 | MH550483 | *Fusarium* ([AF310976](https://unite.ut.ee/bl_forw.php?id=108135)) | 96 | 1480 | 0 |
| daef14 | MH550484 | *Fusarium* ([GQ866861](https://unite.ut.ee/bl_forw.php?id=7200)) | 99 | 1570 | 0 |
| daef15 | MH550485 | *Ceratobasidium* ([DQ097889](https://unite.ut.ee/bl_forw.php?id=84428)) | 97 | 1355 | 0 |
| daef16 | MH550486 | *Ceratobasidium* ([JX243936](https://unite.ut.ee/bl_forw.php?id=368131)) | 92 | 1256 | 0 |
| daef17 | MH550487 | *Ceratobasidium* ([JX243936](https://unite.ut.ee/bl_forw.php?id=368131)) | 94 | 1216 | 0 |
| daef18 | MH550488 | *Ceratobasidium* ([AF354091](https://unite.ut.ee/bl_forw.php?id=108614)) | 99 | 1413 | 0 |
| daef19 | MH550489 | *Chaetomiaceae* ([GU055594](https://unite.ut.ee/bl_forw.php?id=6488)) | 96 | 1324 | 0 |
| daef20 | MH550490 | *Chaetomium* ([KT895345](https://unite.ut.ee/bl_forw.php?id=568594)) | 99 | 1660 | 0 |
| daef21 | MH550491 | *Penicillium simplicissimum* ([AB293968](https://unite.ut.ee/bl_forw.php?id=120309)) | 98 | 1624 | 0 |
|  |  | *Penicillium* ([KF367540](https://unite.ut.ee/bl_forw.php?id=403127)) | 98 | 1526 | 0 |
| daef22 | MH550492 | *Penicillium simplicissimum* ([AB293968](https://unite.ut.ee/bl_forw.php?id=120309)) | 96 | 1503 | 0 |
|  |  | *Penicillium chrysogenum* ([KY352035](https://unite.ut.ee/bl_forw.php?id=744580)) | 96 | 1492 | 0 |
| daef23 | MH550493 | *Aspergillus fumigatus* ([KR023997](https://unite.ut.ee/bl_forw.php?id=571833)) | 99 | 1649 | 0 |
|  |  | *Aspergillus udagawae* ([KY808737](https://unite.ut.ee/bl_forw.php?id=818552)) | 99 | 1561 | 0 |
| daef24 | MH550494 | *Talaromyces* ([KP851982](https://unite.ut.ee/bl_forw.php?id=618709)) | 98 | 1548 | 0 |
| daef25 | MH550495 | *Cladosporium* ([KY781771](https://unite.ut.ee/bl_forw.php?id=812208)) | 99 | 1626 | 0 |
|  |  | *Cladosporium cladosporioides* ([KP701933](https://unite.ut.ee/bl_forw.php?id=561659)) | 99 | 1622 | 0 |
| daef26 | MH550496 | *Bionectriaceae* ([HG937118](https://unite.ut.ee/bl_forw.php?id=612037)) | 99 | 1454 | 0 |
| daef27 | MH550497 | *Bionectriaceae* ([JF449884](https://unite.ut.ee/bl_forw.php?id=191724)) | 99 | 1528 | 0 |
| daef28 | MH550498 | *Clonostachys rosea* ([KT215192](https://unite.ut.ee/bl_forw.php?id=614871)) | 99 | 1644 | 0 |
| daef29 | MH550499 | *Clonostachys rosea* ([EU552110](https://unite.ut.ee/bl_forw.php?id=43341)) | 99 | 1570 | 0 |
| daef30 | MH550500 | *Mucor fragilis* ([JQ972062](https://unite.ut.ee/bl_forw.php?id=312817)) | 97 | 1584 | 0 |
|  |  | *Mucor racemosus* ([HQ010438](https://unite.ut.ee/bl_forw.php?id=178262)) | 99 | 1225 | 0 |
| daef31 | MH550501 | *Mucor racemosus* ([HQ010438](https://unite.ut.ee/bl_forw.php?id=178262)) | 91 | 964 | 0 |
| daef32 | MH550502 | *Mucor fragilis* ([JQ972063](https://unite.ut.ee/bl_forw.php?id=312986)) | 92 | 1371 | 0 |
| daef33 | MH550503 | *Mucor hiemalis* ([JQ912672](https://unite.ut.ee/bl_forw.php?id=315427)) | 99 | 1362 | 0 |
|  |  | *Mucor* ([AM901686](https://unite.ut.ee/bl_forw.php?id=130050)) | 99 | 1312 | 0 |
| daef34 | MH550504 | *Mucor fragilis* ([JQ972062](https://unite.ut.ee/bl_forw.php?id=312817)) | 99 | 1608 | 0 |
|  |  | *Mucor racemosus* ([KX146488](https://unite.ut.ee/bl_forw.php?id=733021)) | 99 | 1180 | 0 |
|  |  | *Mucor racemosus* ([FJ582639](https://unite.ut.ee/bl_forw.php?id=30433)) | 99 | 1180 | 0 |
| daef35 | MH550505 | *Trichoderma koningii* ([AJ301990](https://unite.ut.ee/bl_forw.php?id=141882)) | 97 | 1633 | 0 |
|  |  | *Trichoderma atroviride* ([HQ115671](https://unite.ut.ee/bl_forw.php?id=178222)) | 98 | 1618 | 0 |
|  |  | *Trichoderma hamatum* ([KM491888](https://unite.ut.ee/bl_forw.php?id=481990)) | 99 | 1550 | 0 |
| daef36 | MH550506 | *Trichoderma koningii* ([AJ301990](https://unite.ut.ee/bl_forw.php?id=141882)) | 98 | 1575 | 0 |
|  |  | *Trichoderma asperellum* ([KF723005](https://unite.ut.ee/bl_forw.php?id=429189)) | 99 | 1572 | 0 |
| daef37 | MH550507 | *Myrothecium roridum* ([AJ301994](https://unite.ut.ee/bl_forw.php?id=141887)) | 99 | 1716 | 0 |
| daef38 | MH550508 | *Ijuhya vitelline* ([KY607532](https://unite.ut.ee/bl_forw.php?id=836921)) | 90 | 1173 | 0 |
| daef39 | MH550509 | *Ijuhya vitelline* (KY607535) | 90 | 1200 | 0 |
| daef40 | MH550510 | *Leptosphaeria* ([LC150823](https://unite.ut.ee/bl_forw.php?id=597713)) | 99 | 1635 | 0 |
| daef41 | MH550511 | *Leptosphaeria* ([AB752251](https://unite.ut.ee/bl_forw.php?id=408379)) | 99 | 1393 | 0 |
| daef42 | MH550512 | *Leptosphaeria* ([LC150823](https://unite.ut.ee/bl_forw.php?id=597713)) | 99 | 1602 | 0 |
| daef43 | MH550513 | *Phoma medicaginis* ([EU167575](https://unite.ut.ee/bl_forw.php?id=213022)) | 98 | 1537 | 0 |
| daef44 | MH550514 | *Phoma sojicola* ([EU167568](https://unite.ut.ee/bl_forw.php?id=213029)) | 98 | 1543 | 0 |
| daef45 | MH550515 | *Phoma sojicola* ([EU167568](https://unite.ut.ee/bl_forw.php?id=213029)) | 98 | 1544 | 0 |
| daef46 | MH550516 | *Helotiales* ([LN901106](https://unite.ut.ee/bl_forw.php?id=551054)) | 96 | 1245 | 0 |
|  |  | *Helotiales* ([LN901105](https://unite.ut.ee/bl_forw.php?id=551055)) | 96 | 1234 | 0 |

Fungi were grouped into OTUs defined by 90% internal transcribed spacer (ITS) sequence similarity.
